# Transcriptome Analysis of Spermatogenically Regressed, Recrudescent and Active Phase Testis of Seasonally Breeding Wall Lizards *Hemidactylus flaviviridis*

**DOI:** 10.1371/journal.pone.0058276

**Published:** 2013-03-11

**Authors:** Mukesh Gautam, Amitabh Mathur, Meraj Alam Khan, Subeer S. Majumdar, Umesh Rai

**Affiliations:** 1 Comparative Immuno-Endocrinology Laboratory, Department of Zoology, University of Delhi, Delhi, India; 2 Cellular Endocrinology Laboratory, National Institute of Immunology, Aruna Asaf Ali Marg, New Delhi, India; 3 Department of Physiology, All India Institute of Medical Sciences, New Delhi, India; University of Hyderabad, India

## Abstract

**Background:**

Reptiles are phylogenically important group of organisms as mammals have evolved from them. Wall lizard testis exhibits clearly distinct morphology during various phases of a reproductive cycle making them an interesting model to study regulation of spermatogenesis. Studies on reptile spermatogenesis are negligible hence this study will prove to be an important resource.

**Methodology/Principal Findings:**

Histological analyses show complete regression of seminiferous tubules during regressed phase with retracted Sertoli cells and spermatognia. In the recrudescent phase, regressed testis regain cellular activity showing presence of normal Sertoli cells and developing germ cells. In the active phase, testis reaches up to its maximum size with enlarged seminiferous tubules and presence of sperm in seminiferous lumen. Total RNA extracted from whole testis of regressed, recrudescent and active phase of wall lizard was hybridized on Mouse Whole Genome 8×60 K format gene chip. Microarray data from regressed phase was deemed as control group. Microarray data were validated by assessing the expression of some selected genes using Quantitative Real-Time PCR. The genes prominently expressed in recrudescent and active phase testis are cytoskeleton organization GO 0005856, cell growth GO 0045927, GTpase regulator activity GO: 0030695, transcription GO: 0006352, apoptosis GO: 0006915 and many other biological processes. The genes showing higher expression in regressed phase belonged to functional categories such as negative regulation of macromolecule metabolic process GO: 0010605, negative regulation of gene expression GO: 0010629 and maintenance of stem cell niche GO: 0045165.

**Conclusion/Significance:**

This is the first exploratory study profiling transcriptome of three drastically different conditions of any reptilian testis. The genes expressed in the testis during regressed, recrudescent and active phase of reproductive cycle are in concordance with the testis morphology during these phases. This study will pave the way for deeper insight into regulation and evolution of gene regulatory mechanisms in spermatogenesis.

## Introduction

Reptiles hold a phylogenically crucial place in animal evolution as they are ancestor to both birds and mammals. Exhibition of amniotic membrane places reptiles in close proximity with birds and mammals [1]. Tubular form of spermatogenesis, where spermatogenesis takes place in seminiferous tubule and myoid peritubular cells appear for the first time in reptilian testes [2]. These are the features found in testis of higher order mammals also. These features are maintained in higher order mammals also. The wall lizard is a seasonally breeding animal exhibiting a prenuptial cycle of sperm development [3]. Prenuptial reptiles produce sperm prior to or during the mating season. Reproductive cycle of wall lizard is clearly divided in three phases, regressed from late May to August, recrudescent September-November, and spermatogenically active phase from December to early May [4]. In the recrudescent phase, proliferating spermatogonia and meiotic germ cells are present; in active phase advanced germ cells undergoing spermiogenesis and fully mature sperm supported by mature Sertoli cells are present. While in regressed phase, only quiescent spermatogonia and retracted Sertoli cells are present for long time retaining of their ability to repopulate & redifferentiate. Since, any defect in onset and/or reinitiation of spermatogenesis may lead to testicular condition similar to certain forms of infertility. This periodic subtle transition between spermatogenically active and inactive state of testis in this reptile model offer an appropriate situation to understand over and under expression of genes involved in initiation of mitosis, meiosis, maintenance of sperm production, and finally, seizure of spermatogenic process, which is otherwise not possible in mice or rat.

In mice, microarray has been used as a powerful technique to construct gene regulatory networks for a global view of varied states of testes in different age groups [5]. With the advent of microarray technology, the comparison of mRNA expression by germ cells and hormone-induced gene expression in testes has been addressed [6]. Microarray studies have helped in the identification of novel candidate genes (previously non-documented) that play crucial role in the regulation of spermatogenesis [7]. In light of this, an attempt was made to profile genes expressed during regressed, recrudescent, and active state of testis of wall lizard by employing microarray technique. Although South American green lizard *anole* genome has been sequenced recently [8], microarray chips for the same are not yet commercially available. Because of this constrain and to study genes conserved in lizard and mice, we have used mouse whole genome gene array chips. The present investigation in wall lizard, *H. flaviviridis* is the first exploratory study documenting gene expression across all the phases of its reproductive cycle. Further functional studies based on this microarray data will provide deeper insight in to the specific role of these genes in regulation of spermatogenesis. Although there are several forms of idiopathic male infertility which are untreatable, men do not show distinct demarcation in ability of testis and its recrudescence, hence do not provide opportunity to identify genes important for switching on or off during spermatogenesis.

## Materials and Methods

### Ethics Statement

The guidelines of the “Committee for the Purpose of Control and Supervision of Experiment on Animals CPCSEA,” Government of India, were followed in handling, maintenance, and sacrifice of animals with Institutional Animal Ethics Committee IAEC approval DUZOO/IAEC-R/2011/17.

### Animals Maintenance and Tissue Collection

The wall lizards *Hemidactylus flaviviridis* were procured from in and around the city of Delhi, India. Male animals were housed in wooden cages and acclimatized to laboratory conditions 12L: 12D at least for 8 days before the commencement of the experiments. They were provided food and water *ad libitum*. On an average, 4–6 animals were sacrificed in spermatogenically active phase, 6–8 in recrudescent and 50–60 in regressed phase. The decapsulated testes were snap frozen in liquid nitrogen and crushed to powder with sterile mortar and pestle. The powdered tissue was resuspended in 2 ml of RNA-Save, Biological Industries, Israel, distributed 1 ml each in screw capped sterile tube and kept at 4°C for 4–5 h to get RNA SAVE permeabilized and stored at −80°C until used for RNA isolation. For routine histology, 2–3 testes from each phase were fixed in Bouin’s fixative.

### Histopathology

For evaluating the status of spermatogenesis, testes obtained from various phases of reproductive cycle from wall lizard were fixed in Bouin’s fluid and paraffin blocks were prepared. Sections 4 µm thick were cut with a Reichert Jung microtome 1640 and hematoxylin eosin staining was done following standard procedure [9]. After mounting in DPX mountant, the sections were observed under bright field illumination with an upright microscope, Nikon, MICROPHOT-FX.

### Extraction, Quantification and Quality Control of RNA

Total RNA was isolated from tissue samples using Qiagen RNAEasy Mini kit, Qiagen, Valencia, CA, following the instructions of the manufacturer. Equal amount of crushed tissue from each phase of testis was used for RNA isolation. The yield and purity of extracted RNA were assessed using standard protocols of taking absorbance ratio between 260 nm and 280 nm, and in 1% agarose gel electrophoresis. RNA integrity of individual samples was also assessed by Bioanalyzer 2100, using RNA 6000 Nano Lab Chip, Agilent Technologies Inc., Palo Alto, CA. The algorithm of Agilent 2100 Expert Software automatically calculates RNA Integrity Number RIN for the assessment of total RNA quality based on electropherogram output. RIN score provided a quantitative value for RNA integrity that facilitated the standardization of quality interpretation. Total RNA was considered to be of good quality when the rRNA 28S/18S ratios were greater than or equal to 1.5, with the rRNA contribution being 30% or more and an RNA integrity number RIN was ≥8.0 out of maximum scoring of 10 [10].

### cRNA Synthesis, *In vitro* Transcription, Labelling and Microarray Hybridization

Individual RNA samples from each sub-groups and conditions having RIN scores >8.0 were subjected to whole transcriptome array experiment using the Agilent Whole genome Mouse 8×60 K format Array chip, AMADID: 26986, according to the manufacturer’s recommendations. See table S1 for the subject details of the selected samples. The probe synthesis, hybridization, and post-hybridization stringency wash were performed as described by manufacturer’s protocol Agilent Technologies http://www.genomics.agilent.com. Briefly, both first and second strand cDNA were synthesized by incubating 500 ng of total RNA with 1.2 µl of oligo dT-T7 Promoter Primer in nuclease-free water at 65°C for 10 min followed by incubation with 4.0 µl of 5× First strand buffer, 2 µl of 0.1 M DTT, 1 µl of 10 mM dNTP mix, 1 µl of 200 U/µl MMLV-RT, and 0.5 µl of 40 U/µl RNaseOUT, at 40°C for 2 hour. Immediately following cDNA synthesis, the reaction mixture was incubated with 2.4 µl of 10 mM Cyanine-3-CTP (Perkin-Elmer, Boston, MA), 20 µl of 4X Transcription buffer, 8 µl of NTP mixture, 6 µl of 0.1 M DTT, 0.5 µl of RNaseOUT, 0.6 µl of Inorganic pyrophosphatase, 0.8 µl of T7 RNA polymerase, and 15.3 µl of nuclease-free water at 40°C for 2 hour. Qiagen’s RNeasy mini spin columns were used for purifying amplified cRNA samples. The quantity and specific activity of cRNA was determined by using NanoDrop ND-1000 UV-VIS Spectrophotometer version3.2.1. Samples with specific activity >8 were used for hybridization. 825 ng of each Cyanine 3 labeled cRNA in a volume of 41.8 µl were combined with 11 µl of 10× Blocking agent and 2.2 µl of 25× Fragmentation buffer Agilent, and incubated at 60°C for 30 minutes in dark. The fragmented cRNA was mixed with 55 µl of 2× Hybridization Buffer Agilent. About 110 µl of the resulting mixture was applied to the Agilent Mouse Whole Genome 60 k Array chip Agilent Technologies, and hybridized at 65°C for 17 hours in an Agilent Microarray Hybridization Chamber SureHyb: G2534A in rotating Hybridization Oven. After hybridization, array slides were washed with Agilent Gene expression Wash Buffer I for 1 minute at room temperature followed by a 1 min wash with Agilent Gene expression Wash Buffer II at 37°C. Slides were finally rinsed with Acetonitrile for cleaning up and drying.

### Microarray Data Processing

#### Array analysis

Microarray experiments with individual samples were performed according to standard MIAME guide lines http://www.mged.org/Workgroups/MIAME/miame.html. Hybridized arrays were scanned at 5 µm resolution on an Agilent DNA Microarray Scanner G2565BA. Data extractions and quantification from images were done using Feature Extraction software Agilent Technologies. The extracted raw data were analyzed using GeneSpring Gx v 11.0.1 software from Agilent Technologies. Within each hybridization panel the 50th percentile of all measurements was used as a positive control of normalization for each gene. Per-membrane and per-gene intensity dependent normalization, two-way normalization, lowess normalization, averaging, exploratory analysis and significant ratio analysis were performed on log transformed data using GeneSpring v.11.0.1 Agilent Technologies, Santa Clara, CA, USA. Pearson’s correlation coefficients done to assess the reliability of data obtained from two separate hybridization runs for same RNA preparations and confirmed the reproducibility assurance P<0.01 among hybridizations. Analysis of the data retrieved from separate chips with the same RNA samples yielded QC statistics highly concordant with that of the manufacturer, and it revealed more than 95% confidence level.

#### Cluster analysis

It has been observed that there are thousands of genes for each observation in gene expression data and a few genes or group of gene may account much of the variation in whole data. Principal component analysis PCA is an unsupervised multivariate analysis tool that reduces the dimensionality of the data set by transforming to a new set of variables principal components, PCs and summarizes the data features [11]. The normalized and filtered data were analyzed to characterize the global relationships of individual samples by unsupervised principal component analysis PCA and the exposed clustered groups were displayed in a three dimensional 3-D graph. The PCA algorithm in GeneSpring11.5.1 was applied to all annotated regressed, recrudescent and active Phase samples for all expressed genes to evaluate the similarity in gene expression patterns on the basis of underlying variability and cluster structures. Since PCA reduces data complexity in a rational way without any prior knowledge of the categories, it was used to determine if any intrinsic clustering or outliers existed within the data set [12].

To further evaluate the patterns of gene expression profiles in each sample belonging to different groups and/sub-groups, unsupervised hierarchical clustering of all samples using a standardized Pearson’s uncentered correlation vector with average linkage for distance measures was performed [13]. Sample to sample expressional relationship and visualization in the form of dendrogram and heatmap respectively were yielded in hierarchical cluster analysis. While in *K*-means cluster analysis, gene expression levels were randomly assigned into distinct clusters and the average expression vector was computed for each cluster [13]. For every gene, the algorithm then computed the distance to all expression vectors, and moved the gene to the cluster whose expression vector was closest to it. The entire process was repeated iteratively until no gene products could be reassigned to a different cluster. The whole gene classified into different sets of clusters, Ks clusters, according to their expressional patterns and based on their average expression pattern in different samples, clusters of genes also identified as co-expressed genes.

#### Differential gene expression analysis

The fold-change is a measure of differential expression “signal”, whereas t-statistic is a signal standardized by the noise level, i.e., “signal-to-noise” ratio. The fold-change is an example of absolute effect size, whereas t-test a relative effect size. To maximum utilization of statistical information from the data, fold-change and t-statistic can be displayed simultaneously by volcano plots. Volcano plots allow easy comparison between the “double filtering” gene selection criterion and “single filtering” or “joint filtering” criteria.

Normalized microarray data were subjected to one-way analysis of variance followed by pair-wise comparisons of expression values for each gene between active, recrudescent and regressed phase of reproductive cycle samples respectively. The resulting differential gene lists from each pair-wise comparison only included those genes that showed a fold change of >2.0 or higher and a P<0.05 by using a parametric Welch t test with Benjamini-Hochberg multiple testing corrections for false discovery rate FDR [14]. All statistical analysis was performed using GeneSpring 11.0.1 software.

#### Gene ontology based functional analysis

The gene ontology GO based functional enrichment analysis was performed by using the co-expressed genes of three different clusters K1, K2, and K3 identified in previous K-means cluster analysis [15]. Further networks and different functional enrichment analyses were done using gene lists obtained from the differential gene expression analyses between different sub-groups based on pre-fixed setting of a cut-off threshold of pFDR p≤0.05 with the help of the GeneSpring11.0.1 software, GeneGo Metacore software Thomson Reuters, St. Joseph, MI, USA, DAVID tool http://david.abcc.ncifcrf.gov [16] and the Kyoto Encyclopedia of Genes and Genomes KEGG platform http://www.genome.jp/kegg/for pathways analysis to link genomic information with higher order functional information.

### Quantitative Real-Time Polymerase Chain Reaction

Microarray data was validated by assessing the relative expression of some of the selected genes using SYBR green chemistry by Q-PCR as described earlier [17]. The expression of coronin, actin binding protein 1A **Coro1a**, Secreted acidic cysteine rich glycoprotein **sparc,** Inhibitor of growth family, member 1 **Ing1**, ***Vasohibin VASH1,*** Kringle containing transmembrane protein 1 ***Kremen 1,*** Casein kinase 2, alpha 1**Csnk2a1** and **fibronectin Fn** was checked. The relative quantity was derived from the formula: Fold Change  = 2^−ΔΔCt^. For each sample, the calculated quantity was normalized with relation to the quantity found for GAPDH. The list of primers used in this study is given in **Table 1**.

**Table 1:** List of primers used in this study for the validation of microarray data by Q-PCR.

Gene Name	Primer Sequence*		Primer length	Tm°C	Amplicon Size in base pairs	Accession No.
Csnk1e	F	CAGTGTTGTATGGGGCTTT	19	59.4	131	NM_013767.6
	R	ACAGTCACACAAGGCATCAT	20	58.7		
Fn1	F	GACAACTGCCGTAGACCTG	19	59.3	140	NM_010233.1
	R	TCTAGCGGCATGAAGCAC	18	61.0		
Sparc	F	TGGAGTTAGGCAGAGGGAAGT	21	64.9	139	NM_009242.4
	R	GCTCTGGATTATTCTGTCTTTC	22	60.0		
Coro1A	F	CTCAAGGATGGCTACGTGC	19	62.5	129	NM_009898.2
	R	CTCCAGCCTTGACACGGTA	19	62.6		
VASH1	F	CTGGGATGAGTTTGGGCT	18	61.2	119	NM_177354.4
	R	AATACCCTTGCCCTTCACA	19	61.7		
Ing1	F	GTTAGCCGTGCCTCCTCTC	19	63.5	139	NM_011919.4
	R	CTGACACTCGCTGACATGGT	20	62.1		
Kremen1A	F	GGTAAGCCGAGGGAGAAGAG	20	64.9	132	NM_032396.3
	R	TGTGGGAGAGGATGAAGACC	20	62.9		

* F; Forward primer, R; reverse primer.

## Results

### Evaluation of the Status of Spermatogenesis in the Wall Lizard Testis from Regressed, Recrudescent and Active Phase of Reproductive Cycle

**Figure 1:**
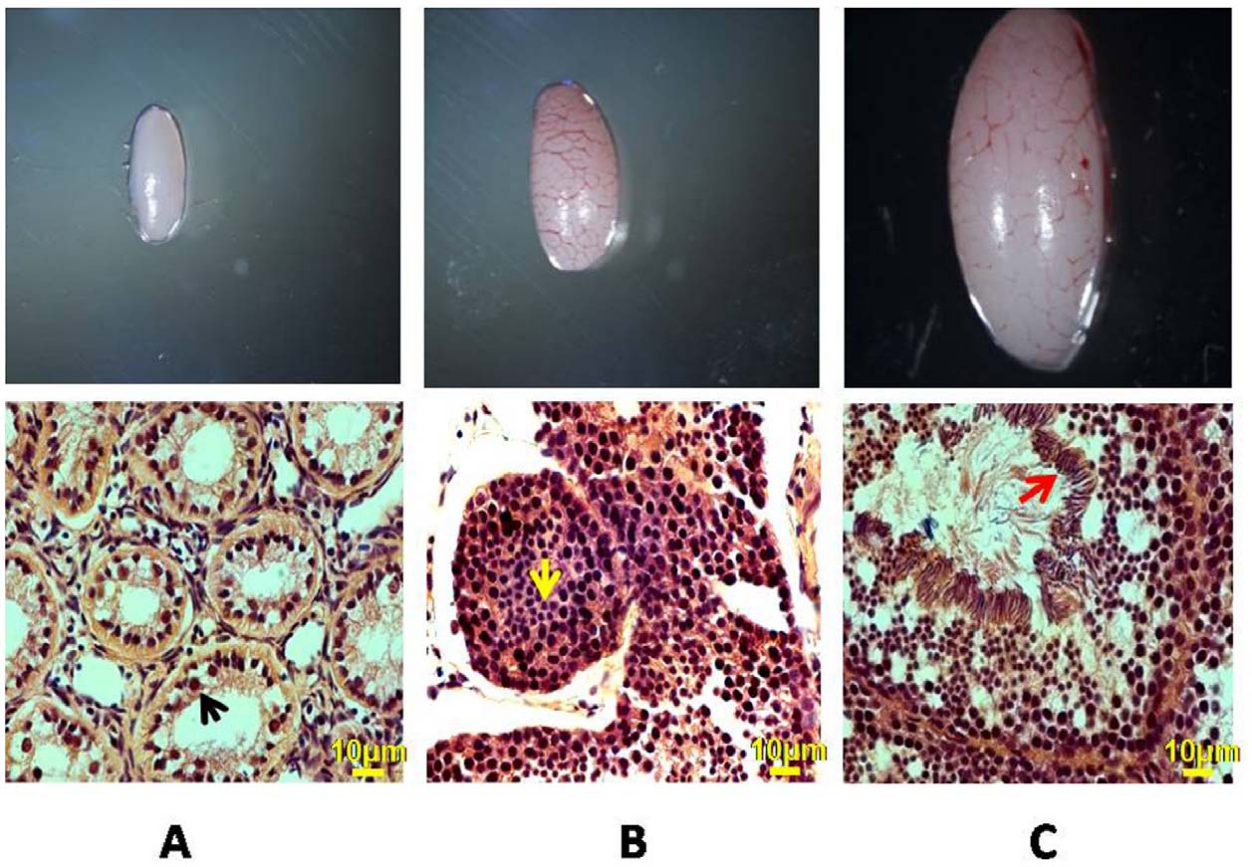
Size and cross section of wall lizard testis. **A** Very small seminiferous tubules, retracted Sertoli cells and only spermatognia are seen in regressed phase testis, black arrow. **B** Testis from recrudescence phase shows large seminiferous tubules, normal Sertoli cells and advanced germ cells, yellow arrow. **C** Testis from active phase is largest in size and sperm in seminiferous lumen can be noticed, red arrow. Bar = 10 µm. Note A = active phase; B = recrudescent phase and C = regressed phase.

Weight and size of wall lizard testis differed significantly during various phases of reproductive cycle. The testis weighed 4–6 mg, 20–25 mg and 36–40 mg during regressed phase, recrudescent phase and active phase respectively. Gonado-somatic Index GSI during regressed, recrudescent and active phase was 5.4×10^−4^, 1.93×10^−3^ and 3.4×10^−3^ respectively. Light microscopic analysis of testicular sections from regressed, recrudescent and active phase of reproduction showed various phases of reproductive cycle. In the regressed phase seminiferous tubules shrunk to the minimal size with no defined tubule morphology. In this phase there are only retracted Sertoli cells and spermatogonia in the seminiferous tubule **Figure 1A**. No other stages of germ cells were seen in this phase. In the testis from recrudescent phase seminiferous tubule size enlarged with full size Sertoli cell and advanced meiotic germ cells. Elongated spermatids embedded in Sertoli cell cytoplasm were also seen in this phase **Figure 1B**. In the testis from active phase, diameter of the tubule became maximal. Sertoli cells were extended holding all the stages of developing germ cells. Sperm head were seen encrypted in the Sertoli cell cytoplasm **Figure 1C**. A detailed comparative description of all the phases of reproductive cycle is given in **Table 2**.

**Table 2:** Description of the different reproductive phases of wall lizard testis.

ReproductivePhase	Description	Durationfrom – to	Testis Weight	Animal Body weight	Gonado-Somatic IndexGSIWeight of testis/Weight of animal
**Regressed or Quiescent**	Retracted Sertoli cell, presence of spermatogonia only, small testis size, shrunk seminiferous tubule	Late May – August	5.0 mg	9.3 gm	5.4×10^−4^
**Recrudescent**	Normal Sertoli cells and testis size, meiotic germ cells, normalseminiferous tubule	September – October	22.5 mg	11.7 gm	1.9×10^−3^
**Active**	Same as recrudescent exceptpresence of mature sperm	November – April	38.0 mg	10.9 gm	3.4×10^−3^

### Microarray Analyses of Wall Lizard Whole Testis from Regressed, Recrudescent and Active Phase of Reproductive Cycle

The data from duplicate array, separate chips arrays with the same RNA samples, yielded high concordant with more than 95% confidence level with that of the manufacturer. The data from 6 samples, 2 samples from each active, recrudescent and regressed phase, which passed quality control parameters, has been deposited to NCBI via the Gene Expression Omnibus GEO data repository http://www.ncbi.nih.gov/geo/vide accession numbers GSE36505. The data deposited to NCBI-GEO can be accessed at http://www.ncbi.nlm.nih.gov/geo/query/acc.cgi?token=njcjxawaaewmuri&acc=GSE36505.

### Cluster Analysis

#### Principal component analysis PCA

**Figure 2:**
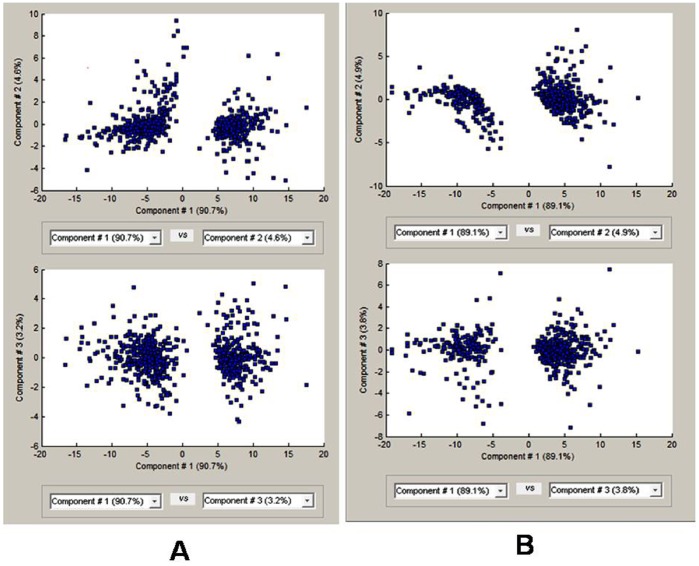
Principal Component Analysis. The PCA of genes differentially expressed between active and regressed phase. Upper panel shows PCA between component 1 and component 2 whereas lower panel shows PCA between component 1 and component 3 shown in **A,** while PCA of genes differentially expressed between recrudescence and regressed phase shown in **B.** Upper panel shows PCA between component 1 and component 2 whereas lower panel shows PCA between component 1 and component 3.

PCA is a mathematical tool that aggregates the correlated samples into new sets of variables/samples using the variability in the gene expression data. Unsupervised PCA based on the percentage variance in gene expression of the active, recrudescence and regressed phase samples categorized into 2 main clusters. **Figure 2** shows the graphical presentation of PCA. The distribution of genes differentially expressed between active and regressed phase in components 1, 2 and 3, while in between recrudescence and regressed phase in components 1, 2 and 3, revealed considerable degree of expression homogeneity in their corresponding groups.

#### Hierarchic clustering analysis HCA

**Figure 3:**
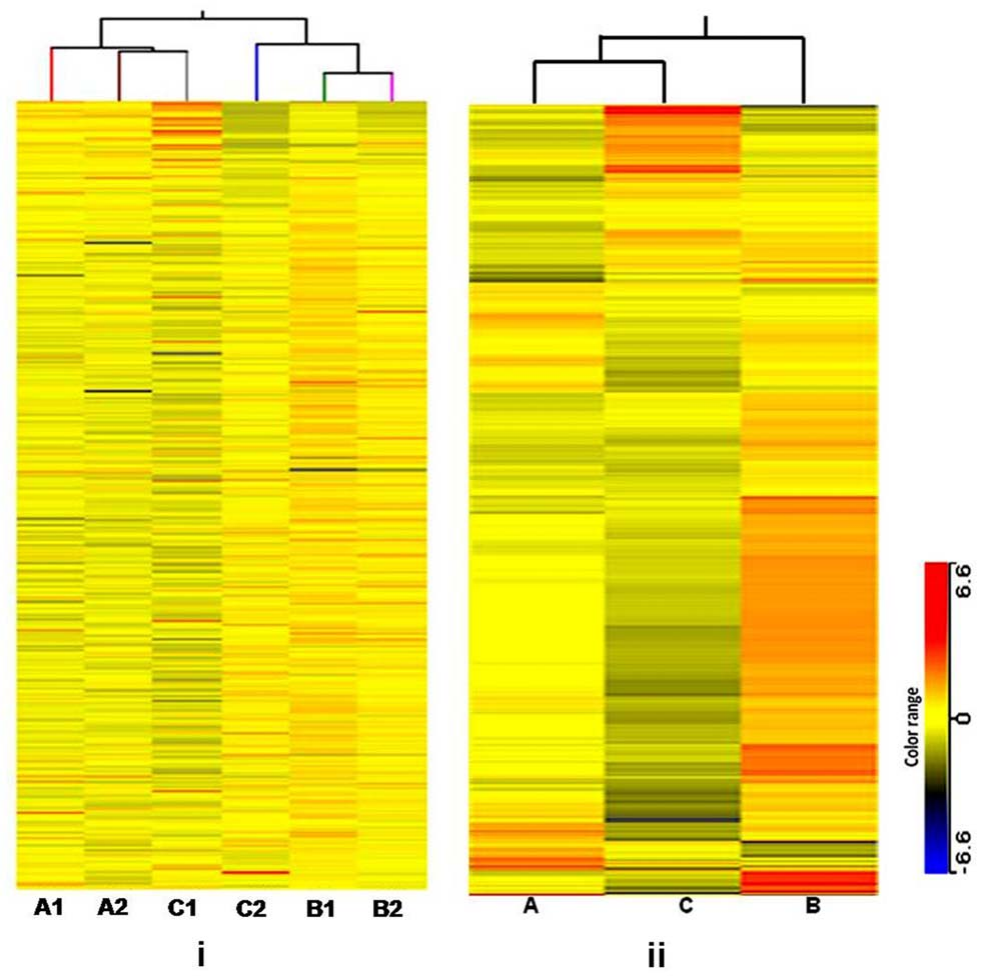
Unsupervised hierarchical clustering. The two-way representation of unsupervised HCA of the expression levels, in logarithmic scale, of all the target probes/genes, Y-axis, in each sample, each column, and their clustering based on expressional distance Pearson correlation coefficient between samples in dendrogram formation, X-axis. Heat map shows the gene expression pattern in defined colour range. **i** Represents the unsupervised HCA of the all 6 samples, 2 samples each from active, recrudescence and regressed phases and **ii** showed the semi-unsupervised hierarchical clustering analysis and heat map of average expression of the replicates in all three groups. Note A = active phase; B = recrudescent phase and C = regressed phase.

The results of the unsupervised HCA of the all 6 samples, 2 samples each from active, recrudescence and regressed phases, and semi-unsupervised hierarchical clustering analysis and heat map showed that gene expression pattern in active phase clustered closely with those in regressed phase and both of these clustered together with recrudescent phase samples. The heat map generated based on HCA showed that there was no significant variation among biological replicates **Figure 3**. This analysis confirmed that the expressions of biological replicates were well correlated with each other.

#### K-means cluster analysis

**Figure 4:**
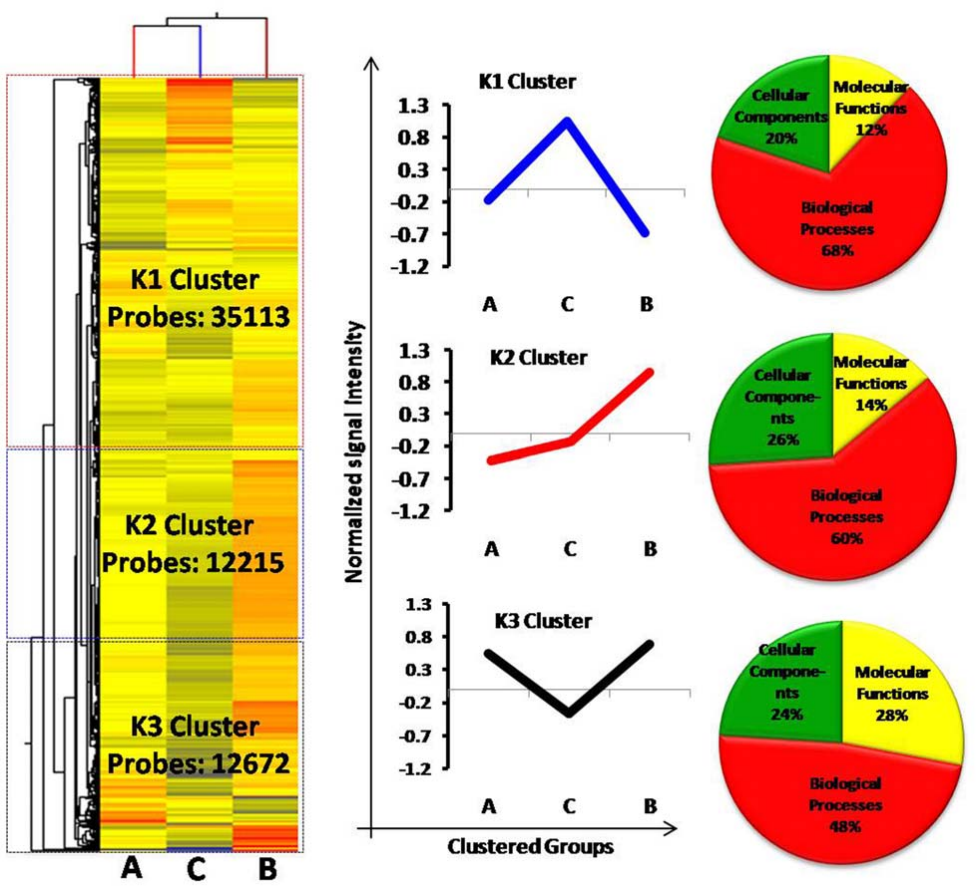
Entity based *K*-mean cluster analysis of entire 60 K probe sets. Entire data was divided in 3 clusters namely K1, K2 and K3. The trend of average expression has been shown by bar graph and Gene Ontology GO analysis has been represented by pie charts describing distribution of data enriching biological processes, molecular functions and cellular components. Note A = active phase; B = recrudescent phase and C = regressed phase.

To identify the clusters of genes whose expression is regulated in a similar way throughout the samples, *K*-means entity based clustering tool was applied to all the expressed genes across all the 6 samples of regressed, recrudescent and active phase testis. As shown in **Figure 4** **,** 3 major groups named as clusters K-1, K-2 and K-3 were identified. The clusters distinguished themselves according to the percentage enrichment of gene ontology GO-based functionality with regard to biological processes, molecular functions, and cellular localization. Out of 60 K probe sets, the largest cluster cluster K-1 consisted of 58.5%, 35113 probes in which expression was highest in regressed phase. Of 35113 probe sets in cluster K-1, 4216, 12% were related to molecular functions, 23890, 68% to biological processes and 7027, 20% to cellular components. The probes of cluster K-1 were over represented in regressed phase. Cluster K-2 included 20.4%, 12215 probes whose expression was gradually increased from active to recrudescence phase. GO functionality showed that out of 12215 probes, 1710, 14% were related to molecular functions, 7329, 60% to biological processes and 3176, 26% to cellular components. These functions showed highest expression in recrudescent phase. Cluster K-3 contained 21.1%, 12672 probes out of which 3548, 28% were associated with molecular functions, 6083, 48% with biological processes and 3041, 24% with cellular components in GO functionality groupings.

### Gene Filtering

**Figure 5:**
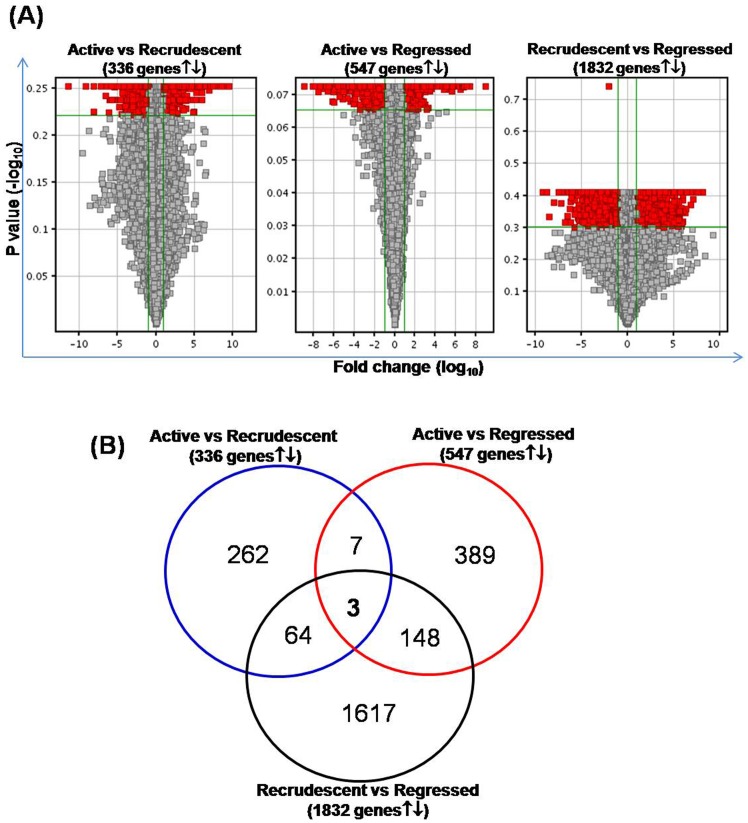
Volcano plot and Venn analysis of differentially expressed genes. **A** Volcano plot, whose X axis represents the fold change, log transformed, and Y axis, the p value, log transformed. If the intensity ratio of a gene in between control and target is more than 2 fold or less than 2 fold having p value less than 0.05, considered as a differentially expressed gene. The genes lying in red colored area are satisfying the differential change criteria and are identified as differentially expressed genes in between two conditions. **B** Venn diagram representing the distribution of differentially expressed, Fold change ≥2 and p-value ≤0.05, genes obtain in active *Vs* recrudescent phase, active *Vs* regressed phase and recrudescent *Vs* regressed phase testis samples analysis. Note that the areas shown in distribution analysis are not strictly in scale.

Testis from regressed phase were considered as control group while recrudescence and active testis were considered as test groups. As shown in Volcano plot and Venn diagram **Figure 5** total of 336 genes were differentially regulated in active *Vs* recrudescence group analysis, of which 74 and 262 genes showed the common and exclusive differential expression with other analyzed groups respectively. Total 547 differentially regulated genes were identified in active *Vs* regressed group analysis, of that 158 and 389 genes showed the common and exclusive differential expression with rest of the analyzed groups respectively. Highest number of genes, 1832, found differentially regulated in recrudescence *Vs* regressed phase analysis, of which 215 and 1617 genes showed the common and exclusive differential expression with rest of the analyzed groups respectively. 7 genes, *Ppib, H3f3b, Aass* and *Gm5848* were down regulated and *Prh1*, *Apob* and *Olfr129* were up regulated, were commonly regulated in between both recrudescence and regressed phase compared with active phase testis samples. 148 genes were commonly regulated in between active *Vs* regressed *and* recrudescence *Vs* regressed phase samples, while 64 genes showed the common expression in between active *Vs* recrudescence and recrudescence *Vs* regressed phase samples. Of all, only 3 genes, *Gm2800, Fam76b* and *Gm11546,* were commonly regulated in all the analyzed groups during three phases. These genes are hypothetical in nature and have not yet been annotated for coding specific protein. The complete list of differentially expressed genes in each group is given in supplementary Tables **S2, S3, S4, S5, S6**. The differentially regulated genes having least p values and higher fold changes in between active, recrudescence and regressed phases were further funnel out based on the functional involvement in lizard spermatogenesis and reproduction, important genes are listed in **Table 3**.

**Table 3:** Lists of the differentially regulated genes identified from microarray data in recrudescent and active phase samples compared with regressed phase samples.

GeneName	Probe Set IDAgilent gene chip	Gene ID	Gene Description	Gene expression as compared to Regressed PhaseFold change –log2*	
				Recrudescent Phase	Active Phase
**Aass**	GT_Mm_44k_51_P483544	30956	Aminoadipate-semialdehyde synthase nuclear gene encoding mitochondrial protein	−1.01	−4.34
**Aifm2**	GT_Mm_44k_52_P2800	71361	Apoptosis-inducing factor mitochondrion associated 2 (Aifm2), transcript variant 1	2.51	3.68
**Anapc7**	GT_Mm_44k_51_P442481	56317	Anaphase promoting complex subunit 7	3.40	1.96
**Cdx1**	GT_Mm_44k_51_P318999	12590	Caudal type homeo box 1	0.52	3.68
**Cep63**	GT_Mm_44k_51_P149313	28135	Centrosomal protein 63	3.57	−0.27
**Crnkl1**	GT_Mm_44k_52_P440102	66877	Crooked neck-like 1 (Drosophila)	−5.94	−2.82
**Csnk2a1**	GT_Mm_44k_51_P397768	12995	Casein kinase 2, alpha 1	2.30	4.37
**Ddx1**	GT_Mm_44k_51_P387220	104721	DEAD(Asp-Glu-Ala-Asp)box polypeptide 1	0.30	3.26
**Dhx32**	GT_Mm_44k_51_P285997	101437	DEAH (Asp-Glu-Ala-His) box polypeptide 32	4.02	0.47
**Elavl4**	GT_Mm_44k_51_P503722	15572	ELAV (embryonic lethal, abnormal vision, Drosophila)-like 4 (Elavl4), transcript variant 1	−4.14	−0.78
**Elmo1**	GT_Mm_44k_51_P306129	140580	Engulfment and cell motility homolog (C. elegans) 1	2.06	3.79
**Epc1**	GT_Mm_44k_52_P117197	13831	Enhancer of polycomb homolog 1(Drosophila) (Epc1), transcript variant 2	−1.89	−4.27
**Fat1**	GT_Mm_44k_51_P350252	14107	FAT tumor suppressor homolog 1	−4.03	−4.36
**Gadd45g**	GT_Mm_44k_51_P315904	23882	Growth arrest and DNA-damage-inducible 45 gamma	−3.85	−1.48
**H3f3b**	GT_Mm_44k_52_P61786	15081	H3 histone, family 3B	−1.08	0.27
**Hira**	GT_Mm_44k_51_P125395	15260	Histone cell cycle regulation defectivehomolog A	3.74	3.24
**Ing1**	GT_Mm_44k_51_P463941	26356	Inhibitor of growth family, member 1	5.06	2.39
**Kremen1**	GT_Mm_44k_51_P390239	84035	Kringle containing transmembrane protein 1	2.60	3.52
**Leprotl1**	GT_Mm_44k_52_P395220	68192	Leptin receptor overlapping transcript-like 1	3.12	5.79
**Moap1**	GT_Mm_44k_51_P475291	64113	Modulator of apoptosis 1	0.75	2.36
**Msi2**	GT_Mm_44k_52_P628060	76626	Musashi homolog 2 (Drosophila)	−2.37	−1.36
**Myst1**	GT_Mm_44k_51_P103757	67773	MYST histone acetyltransferase 1	0.46	3.24
**Nfat5**	GT_Mm_44k_51_P135309	54446	Nuclear factor of activated T-cells 5	1.57	2.39
**Pax5**	GT_Mm_44k_51_P122855	18507	Paired box gene 5	3.28	4.79
**Pcdh10**	GT_Mm_44k_52_P78439	18526	Protocadherin 10 (Pcdh10), transcript variant 3	−2.91	−6.58
**Ptch1**	GT_Mm_44k_51_P127435	19206	Patched homolog 1	1.13	4.51
**Ptgir**	GT_Mm_44k_52_P681368	19222	Prostaglandin I receptor	−3.20	−1.18
**Raver1**	GT_Mm_44k_52_P333352	71766	Ribonucleoprotein, PTB-binding 1	1.56	3.15
**Runx2**	GT_Mm_44k_51_P230942	12393	Runt related transcription factor 2	−8.49	−4.86
**Serpine2**	GT_Mm_44k_51_P268094	20720	Serine (or cysteine) peptidase inhibitor,clade E, member 2	5.01	1.36
**Sirt6**	GT_Mm_44k_01331	50721	Regulation 2, homolog) 6 (S. cerevisiae)sirtuin 6 (silent mating type information	−3.53	−0.82
**Slc2a8**	GT_Mm_44k_51_P358171	56017	Solute carrier family 2, (facilitated glucose transporter), member 8	3.61	4.91
**Sp3**	GT_Mm_44k_52_P522264	20687	Trans-acting transcription factor 3 (Sp3), transcript variant 1	−0.15	0.82
**Sycp3**	GT_Mm_44k_01569	20962	Synaptonemal complex protein 3	3.17	4.65
**Trps1**	GT_Mm_44k_51_P240384	83925	Trichorhinophalangeal syndrome I	2.54	3.14
**Tubb1**	GT_Mm_44k_00653	545486	Tubulin, beta 1	4.27	6.51
**Ube2d3**	GT_Mm_44k_52_P229709	66105	Ubiquitin-conjugating enzyme E2D 3(UBC4/5 homolog, yeast)	3.97	5.01

* Negative sign represent that genes are down regulated while others are up-regulated in between analyzed conditions.

### Gene Ontology GO Enrichment Analysis

**Figure 6:**
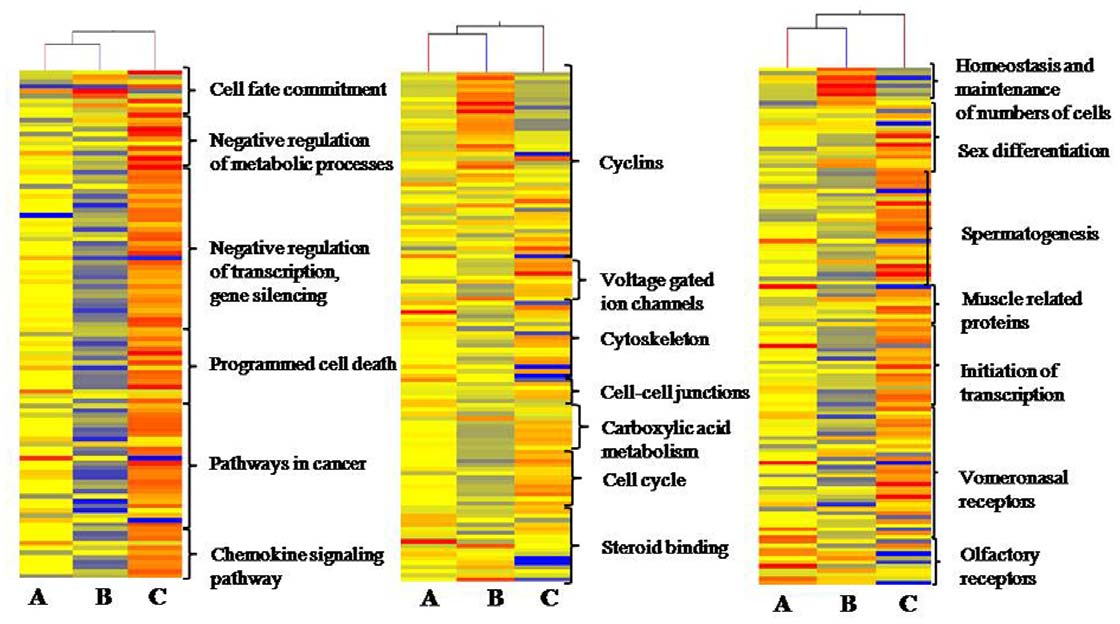
Functional clustering of biologically important genes. Heat map showing changes in expression of genes related to some biologically important functional categories during active, recrudescent and regressed phase of wall lizard reproductive cycle. Heat maps were constructed using average of raw signals for each gene in microarray data.

**Figure 7:**
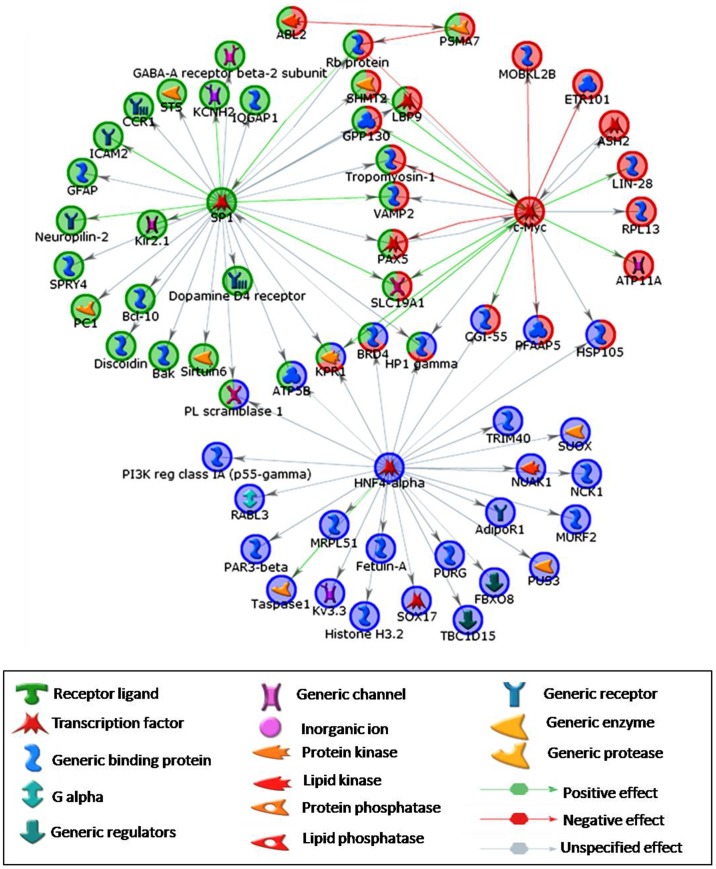
Interactome and networks. Interaction of three transcription factors, HNF-4, c-Myc and Sp1, with other important pathway candidate genes. These three transcription factors are prominently expressed commonly in both recrudescent and active phase.

The GeneGo Metacore, GeneSpring 11.0.1 and DAVID software’s have been employed for Gene Ontology analysis. Further conditional test was used to enrich more specific GO terms. The cohort of the genes identified in pair-wise analysis in between active, recrudescence and regressed phase testis samples were considered for further GO analysis. The GO terms cytoskeleton, cell-cell junctions, transcription, osmo-regulation, differentiation, cell cycle, and niche maintenance showed the involvement during various phases of lizard reproductive cycle **Table 4**. Genes involved in cytoskeleton, cellular growth, apoptosis, initiation of transcription, cell division and regulation of cellular metabolism were over represented in active and recrudescent phase. Genes playing important roles including negative regulation of transcription, stem cell niche maintenance, inhibition of cell growth and negative regulation of metabolism were upregulated in regressed phase **Figure 6**. Three transcription factors, *Sp1, HNF4-α* and *c-Myc* have shown to be prominently expressed in both recrudescent and active phase samples. These transcription factors regulate a number of other important genes involved in downstream signaling cascade. **Figure 7** shows interaction of these transcription factors with other genes.

**Table 4:** Functional category enrichment analysis based on Gene Ontology terms.

Biological Process	GO Term ID	Counts	% of Differentially Regulated genes	p-Value
Cell junction	GO:0030054	8	7.4	0.009
Cellular homeostasis	GO:0019725	6	2.04	0.56
Spermatogenesis	GO:0007283	3	1.02	0.89
Positive regulation of growth	GO:0045927	4	3.3	0.004
Cytoskeleton	GO:0005856	13	10.83	0.02
Steroid binding	GO:0005496	7	0.46	0.06
Positive regulation of cytokinebiosynthetic process	GO:0042108	7	0.46	0.07
Homeostasis of number of cells	GO:0048872	14	0.92	0.05
Germ cell development	GO:0007281	13	0.86	0.05
Gene silencing	GO:0016458	6	0.39	0.03
				
Cell fate commitment	GO:0045165	8	0.53	0.9
Tissue remodeling	GO:0048771	3	0.19	0.85
				
Negative regulation of gene expression	GO:0010629	37	2.4	0.01
				
Cell division	GO:0051301	3	4.5	0.2
Apoptosis	GO:0006915	30	1.9	0.78
Transcription initiation	GO:0006352	3	2.4	0.02
GTpase regulator activity	GO:0030695	37	2.4	0.02
Negative regulation of macromolecule metabolic process	GO:0010605	8	3.9	0.21
Blood vessel morphogenesis	GO:0048514	7	2.3	0.07

% of DR refers to the percent of differentially regulated transcripts falling under the term; p-value is the raw p-value from Fishers exact test. GO term analysis was done using DAVID bioinformatic tool for functional analysis (http://david.abcc.ncifcrf.gov/).

### Validation of Microarray Data by Quantitative Real Time Polymerase Chain Reaction

**Figure 8:**
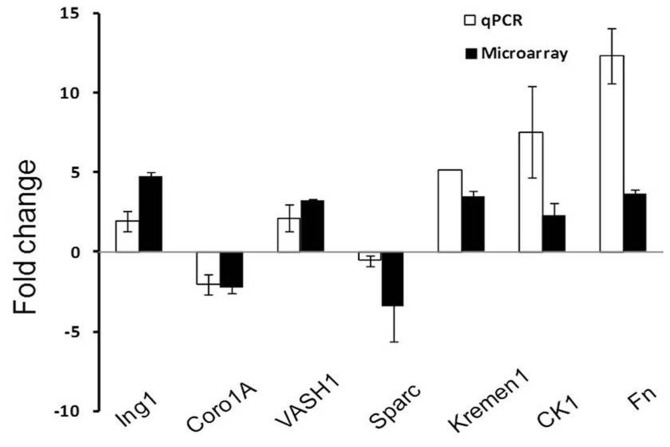
Validation of microarray genes by quantitative Real Time PCR, qPCR. Similar trends with high degree of concordance are represented in between Q-PCR and array data. Filled black bar represent the microarray data while hollow bar is for qPCR data.

The transcripts of some selected differentially expressed genes were further quantified using q RT-PCR for microarray data validation. The relative expressions of 7 genes, *Ing, Coro1A, Vasohibin, Sparc, Kremen 1, Casein Kinase1 and Fibronectin* were validated by quantitative real time PCR in active phase compared with regressed phase testis samples. *Coro1A and Sparc* were down-regulated in active phase as compared to regressed phase, while rests of 5 genes were found to be up-regulated in active phase. The histogram in **Figure 8** represents that all the genes considered for validation showed similar expression profile in both real time PCR and microarray analysis. Although the extent of expression varies between microarray and Real Time PCR data yet the trend remain similar.

## Discussion

Many features of the reptilian testis resemble with mammalian testis. Reptiles are the first group of animals adapted successfully to terrestrial life outside water owing to the ability of internal fertilization. Insightful studies of reptilian reproduction elucidate our understanding of differences between oviparous and viviparous animals [18]. In the evolution, only squamate reptiles and mammals show viviparity [19]. Exhibition of amniotic membrane places reptiles in close proximity to birds and mammals. Unlike amphibians and fishes, lizard testis consists of tubular, seminiferous tubules, and interstitial spaces similar to mammalian testis [20]. Seminiferous tubules are covered with a non-cellular basement membrane and these cells are called as fibroblast/myoid-like peritubular cells Ptc. Ptc are absent in anamniotes and they make their first phyletic appearance in the reptilian testis [2]. Microarray studies on reptilian models are negligible and very limited. This is the first report of high throughput microarray analysis of whole testis of any reptilian organism. PubMed search for “reptilian microarray” on 15.09.2012 returned with only one result in which cDNA microarray was employed to analyze gene expression in Australian snake venom glands [21].

The knowledge of gene sequences is a prerequisite for employing microarray technique to study gene expression [22]. However, use of microarray technique is restricted to few model organism species such as human, rat, mouse etc. because largely their genome has been sequenced making it possible to fabricate microarrays for such organisms [23]. Unfortunately, sequenced genomes are not available for most of the reptilian species; hampering the use of microarray to assess large scale gene expression in these species [18]. The very high cost and huge amount of work involved in developing and producing a DNA array or microarray for a new species is prohibitive for most researchers working in comparative biology. The alternative is to explore the use of heterologous array hybridization, screening for gene expression in one species, using an array developed for another species [24]. Such heterologous or crossspecies microarray hybridization analyses have been executed to study differential gene expression in several non-model species [25]–[32]. It is challenging to study gene expression in developing organs due to its dynamic nature [33]. During a reproductive cycle, the cellular components of wall lizard testis undergo dramatic changes in number, differentiation and transcription in cells. Although whole organ microarray cannot represent cell specific gene expression [34]–[36], it can provide overall gene expression profile in various stages of reproductive cycle, active, recrudescent & regressed phase. The limitations in isolating various cell types from lizard testis, especially during regressed phase, due to its compactness, and small size restricts comparison of cell specific gene expression. However, transcriptome analyses of regressed, recrudescent and active phase testis of wall lizard can describe key changes in gene expression within testis during these phases.

Testes from regressed phase were collected during late June, recrudescent from early October and active phase from late March. Histological analysis and the total RNA obtained from equal amount of tissue from all groups represented physiological activity of testes from different phases of reproductive cycle. 8 fold lesser amount of RNA in regressed testes as compared to that by recrudescent and active phases indicates that in the regressed phase of breeding, testes displays severe quiescence and becomes physiologically inactive **Table S1**. The results of hierarchical clustering analysis, HCA, and K-mean clustering of all samples revealed a high order of sample homogeneity. The resulting gene lists from each pair-wise comparison only included the genes that showed a fold change of 2.0 or higher and a *P*≤0.05 by using a parametric Welch *t* test with Benjamini-Hochberg multiple testing corrections for false discovery rate [14]. The real time RT-PCR based quantitative analysis for expression of 7 target genes from all 6 samples selected on the basis of microarray information validated the array data with a high degree of concordance. *K-*mean entity based cluster analysis divided the entire 60 K genes expression profile in three clusters. The cluster K-1 included largest number of genes 58% whose expression pattern was similar in active and recrudescent phase and higher in regressed phase. In the cluster K-3 there were 23% genes whose expression pattern was opposite to what that was in cluster K-1. In cluster K-3, expression was similar in active and recrudescent phase and was least in regressed phase. Around 20% genes made the cluster K-2 in which there was no significant change in expression of genes between active and regressed phase and expression was highest in recrudescent phase.

During a reproductive cycle, lizard testis shuttles between severe quiescent to active states of spermatogenesis. In regressed phase there is no physiological activity whereas testis resumes activity in recrudescent phase leading to its peak in active phase. This study provides a changing landscape of regressed, recrudescent and active phase testis at gene level. Functional analysis reveals drastic increase in differentially regulated genes in recrudescent phase as compared to regressed phase, which is further maintained in active phase. This change in gene expression is due to reactivation of cellular functions in recrudescent phase. In the recrudescent phase, Sertoli cell regain its shape and proliferate to provide full size to testis. Sertoli-Sertoli cell junctions make blood-Testis-Barrier BTB leading to compartmentalization. Concomitantly, spermatogonia begin proliferation by mitosis to increase in number and meiotically to reach up to elongated spermatid stage [37]. Cell division is an important event in recrudescent and active state testis. Sertoli cells divide mitotically to make optimum number of cells to give final size to the testis and hold appropriate number of germ cells. Germ cells divide both mitotically and meiotically to produce sperm. Active phase is similar to recrudescent phase except the presence of mature sperm in seminiferous tubule lumen. Down regulation of negative regulators of transcription in active and recrudescent phase, meant up regulation of these genes in regressed phase. Functional analysis of differentially regulated genes attributes gene functions during various phases of reproductive cycle. The prominent groups of genes showing change in expression during progression through reproductive cycle are related to cyto-architecture maintenance, apoptosis, transcription, cell division, differentiation, gamete development, DNA repair, cell growth, metabolism, stem cell niche and maintenance of cellular homeostasis. These are discussed below under separate subheadings for better understanding.

### Genes Related to Cyto-architecture

The expression of genes involved in cytoskeleton maintenance *Actg, Actl7b*, *Ccin, Clasp, Dynlt3, Krt17, Ldb3, Nav1, Palld, Ttll3, and Tuba8* and cell-cell junctions *Cadm3, Cldn10a, Ldn19, and Sspn* was profound in recrudescent and active phases. Tubulin [38], [39], actin [40], Palld [41], and Raver1 [42] are implicated in cytoskeleton organization, cellular events such as mitosis, cellular organization, transport, focal adhesions and motility. The claudins are established junctional proteins essential for maintenance of spermatogenic epithelium [43]. Expression of these genes in recrudescent and active phase testis indicated progressive development of cytoskeletal structure bearing large number of differentiating germ cells.

### Genes Related to Apoptosis

Programmed cell death is the prominent feature of spermatogenic testes. Sertoli cell mediated apoptosis is an essential event to maintain a static ratio of germ cells per Sertoli cell [44]. Upregulation of apoptosis related genes *Aifm2, Bcl10, Card10, Elmo1, Fastkd5*, *Pacs2, Sqstm1,* and *Stk4* in recrudescent and active phases testis showed importance of programmed cell death in these phases. *Elmo1* is a pivotal gene involved in Sertoli-cell-mediated removal of apoptotic germ cells [45]. It is also important to note that during regressed phase, no apoptotic activity is inhibited because the germ cell differentiation is at hold.

### Genes Related to Muscle Band

The expression of large number of muscle band proteins *Ank2, Slmap, Spnb2, Smtnl1* and *Ttn* in regressed phase was associated with the occurrence of multilayered peritubular cell over seminiferous tubule. In the recrudescent and active phase, peritubular cells’ layer become single layered. Multiple layers during regressed phase are probably necessary for protecting seminiferous tubule from damage.

### Genes Related to Homeostasis of Number of Cells

Development of cell population in testes is tightly controlled. Genes involved in maintenance of optimum number of cells were *Ireb2, Bpgm, Sox6, Il7r, Coro1a* [46], *Ank1, Lilrb3, Ikbkg, Hoxb6* [47], *Mtap7* [48], *Klf1* [49] upregulated in recrudescent and active phase. These genes determine number of cells required in a tissue or organ by regulating cytoskeleton, cell division, cell-cell interaction and microtubule organization.

### Genes Related to Cell Division and Cell Growth

Controlled cell division and regulated cell growth is essential phenomenon in testis to maintain proper testis size and adequate sperm output. Genes regulating cellular growth, cell cycle and protein biosynthesis were *Ranbp1* [50], [51], *Nedd9* [52], *Ing1* [53], [54], *Sirt2* [55], CEP63 [56], Lig1 [57] and *Fhl* [58], [59]. These genes are important in regulating proliferation, differentiation, adhesion, migration, and signal transduction through interactions with other cellular proteins. Upregulation of these genes in recrudescent and active phase suggested critical role of these genes in the cell division and growth in spermatogenic testis.

Contrary to upregulation of growth and cell division promoting genes in recrudescent and active phase, genes keeping check on cell growth *Nkx3-1, Apc, Ski, Socs5, Lkb1* were upregulated in regressed phase. These genes, *Nkk3.1* [60], [61], *Lkb1* Liver Kinase B1 [62] and *Ski* [63] play an important role in negative regulation of cell growth. Since cell growth and division is completely arrested in regressed phase, these genes are crucial for maintaining quiescent state of testis.

### Genes Related to Transcription

Transcriptional machinery of testis restarts in recrudescent phase of testis after a hiatus of nearly 3 months and maintained in active phase. Active and recrudescent testes are actively engaged in transcription and genes crucial in initiation and regulation of transcription were *Pax5* [64]–[66], *Dhx32* [67], *Meis1* [68] and *Nfi* [69], [70] are up-regulated. These genes play critical role in initiation and maintenance of active transcriptional machinery in the cell. Expression of these genes may allow recrudescence of regressed testis.

Contrary to recrudescent and active phase, regressed phase is the most inactive phase of breeding cycle. In this phase, testis entered in severe quiescence leading to complete cessation of cellular activity. Higher expression of genes involved in transcriptional silencing or negative regulation of gene expression turns off the transcriptional activity in regressed testis. The genes involved in transcriptional silencing were *Cebpa* [71], *Tnrc6b* [72], *Jarid2* [73]–[75] and *Nr2f1* and *Sirt7*. These genes are global transcriptional repressors that balance cell lineage choices during embryonic development and silencing and degradation of the miRNA-targeted mRNAs. *H3f3a* is a transcriptional regulatory gene expressed in all the phases of reproductive cycle with highest expression in regressed phase, indicating its role in controlling regulation of transcription.

### Genes Related to the Maintenance of Niche for Spermatogonial Stem Cell

In the regressed phase testis, only spermatogonia and retracted Sertoli cells are present. Genes involved in cell fate commitment *Smo, Ascl1, Erbb4, Neurod1, Smad2, Sox6, Pitx1*, and *Tlx1* and chemokine signaling pathway *Sos1, Rasgrp2, Ikbkg, Ccl27a, Gnb5, Cxcr4, Il1rap, Il7r, Csf1r, Cnntb1* and cytokine biosynthesis *Icosl, Ereg, Rel, Tbk1, Glmn, Cd209b, Cd27* were up-regulated in regressed phase. All these genes are crucial for maintenance of stem cells in their respective niches and later their differentiation into specialized cell; sperm in case of testis [76]. CXCL12 is expressed by Sertoli cells and its ligand *Cxcr4* is expressed by germ cells [77]. Interaction between the chemokine *Cxcl12, Sdf1* and the G-protein coupled receptor *Cxcr4* is responsible for the maintenance of adult stem cell niches and migration of primordial germ cells. *Cbx3* encoded *Hp1γ* is required for proper germ-cell renewal and survival in the testes [78]. *Lin28b* is a human homolog of lin-28 of nematode *Caenorhabtidis elegans* and is crucial in deriving stem cells from somatic cells [79]. Expression of a constitutively activated form of β-catenin in postnatal Sertoli cells causes male infertility via progressive deterioration of seminiferous tubules, germ cell loss, and testicular atrophy [80]. The transcription factors *Ascl1, Erbb4, Neurod1* plays crucial role in cell fate determination [81], [82]. Upregulation of these genes in regressed phase indicated that spermatogonia are carefully maintained during this dormant phase in their respective niche by Sertoli cells.

### Genes Related to Osmoregulation

Osmo-regulation is an essential function of active testis. Tonicity-responsive enhancer binding-protein *Tonebp* or *Nfat5*, which belongs to the *Rel/Nfat* family of transcription factors, plays a critical role in osmoregulation by controlling the expression of osmoprotective genes [83], [84]. Higher expression of these genes in recrudescent and active phases showed importance of osmo-regulation during normal spermatogenesis.

### Genes Related to Cellular Differentiation

Genes involved in cell differentiation *Fev, Fzd3, Hspa1a, Srpk2, Sqstm1, Ppp3r1, Prm1, Ptch1, Racgap1, Sp1*, and *Sp3* were up-regulated in active phase testis. Many of these genes were part of serine/arginine-rich protein family, Sr proteins, and Sp family of proteins. The *Sr* proteins *Srsf3* and *Srsf1* bind histone H3 tail to control cell cycle progression [85], [86]. Sp1 belongs to a larger family of factors which bind G/C box elements to either activate or repress transcription. The Sp family of proteins is ubiquitous and tissue-restricted transcription factors found to regulate the promoters of several genes, including cell-cycle regulated genes [87], [88]. These genes play important role in cellular differentiation including those of Sertoli cells and germ cells through regulation of cell cycle.

### Conclusion

This study documents the global change in gene expression pattern across different phase of spermatogenesis in the wall lizard, *Hemidactylus flaviviridis,* testis. Data analysis revealed that active and recrudescent phase testis expresses genes involved primarily in “cytoskeleton maintenance”, “apoptosis”, “metabolic pathways”, “transcription initiation” and “cellular growth and differentiation”. Contrary to that, in the regressed phase, the up-regulated genes were mainly involved in stem cell niche maintenance, transcriptional repression, and negative regulation of growth and maintenance of terminally differentiated state.

Regressed phase is a unique condition where cellular activities reach to standstill. Regressed phase may serve as model for infertility where only spermatogonial stem cells are present and require maintaining their potential to proliferate. Detailed analysis of genes regulated in regressed phase may give us clue about causes of such quiescent situation. The genes expressed in recrudescent phase may prove to be crucial to treat such quiescent conditions as in recrudescent phase genes important for re-initiation of cellular activity are expressed. Our data indicates that the genes involved in spermatogonial stem cell niche maintenance were expressed in regressed phase testis of lizard. This shows conservation of genes involved in basic cellular functions in testis from reptiles to mammals. Present study provided knowledge about several group of genes associated with onset of spermatogenesis. In depth studies of functional genomics studies using transgenic mice over expressing or shutting down of functions of these genes selected out of our microarray data will strengthen our knowledge in understanding genes required for governing germ cell differentiation and sperm production.

## Supporting Information

Table S1 **Quantification of total RNA extracted from equal amount of tissue (in duplicate) from Active, Recrudescent and Regressed phase testis of wall lizard.**
(DOC)

Table S2 **Complete list of genes commonly expressed in both active phase and recrudescent phase as compared to regressed phase.**
(XLS)

Table S3 **Complete list of genes expressed only in active phase as compared to regressed phase.**
(XLS)

Table S4 **Complete list of genes expressed only in recrudescent phase as compared to regressed phase.**
(XLS)

Table S5 **Complete list of genes commonly expressed between active phase compared to recrudescent phase and recrudescent phase compared to regressed phase.**
(XLS)

Table S6 **Complete list of genes expressed only in active phase as compared to recrudescent phase.**
(XLS)
