# Supplementary material for: Transcriptome Analysis of Spermatogenically Regressed, Recrudescent and Active Phase Testis of Seasonally Breeding Wall Lizards Hemidactylus flaviviridis
Source: PLoS One. 2013 Mar 11;8(3):e58276. doi: 10.1371/journal.pone.0058276 (PMC3594293; doi:10.1371/journal.pone.0058276)
Supplement: Table S1 — Quantification of total RNA extracted from equal amount of tissue (in duplicate) from Active, Recrudescent and Regressed phase testis of wall lizard. (DOC) [file pone.0058276.s001.doc]

**Table S1:** Quantification of total RNA extracted from equal amount of tissue (in duplicate) from Active, Recrudescent and Regressed phase testis of wall lizard.

| **Sample*** | **Absorbance value 260/280** | **Absorbance value 260/230** | **Concentration**  **ng/ul** |
| --- | --- | --- | --- |
| A1 | 2.90 | 2.07 | 1169.94 |
| A2 | 3.40 | 1.89 | 1372.39 |
| B1 | 1.37 | 2.12 | 548.23 |
| B2 | 1.59 | 2.03 | 639.94 |
| C1 | 0.20 | 1.69 | 83.51 |
| C2 | 0.27 | 1.57 | 109.92 |

*A1, A2 = Active phase; B1, B2 = Recrudescent phase and C1, C2 = Regressed phase.

Note that total RNA extracted from regressed phase is much lesser than recrudescent and active phase.
